# Socioeconomic inequalities in human papillomavirus knowledge and vaccine uptake: evidence from a cross-sectional study in China

**DOI:** 10.3389/fpubh.2024.1399192

**Published:** 2024-06-27

**Authors:** Xiaoqian Gong, Jing Xu, Yuzhen He, Guofang Zou, Jing Liu

**Affiliations:** ^1^Administrative Office, Yuebei People's Hospital, Medical College, Shantou University, Shaoguan, Guangdong, China; ^2^Quality Management Department, Yuebei People's Hospital, Medical College, Shantou University, Shaoguan, Guangdong, China; ^3^Nursing Department, Yuebei People's Hospital, Medical College, Shantou University, Shaoguan, Guangdong, China; ^4^Gynecology Department, Yuebei People's Hospital, Medical College, Shantou University, Shaoguan, Guangdong, China

**Keywords:** HPV vaccine, HPV-related knowledge, inequality, concentration index, China

## Abstract

**Objective:**

Providing the human papillomavirus (HPV) vaccine is effective to eliminate the disparity in HPV-related cancers. It is unknown regarding inequality in the distribution of HPV vaccination in China since the vaccine was licensed and approved for use in 2016. This study aimed to examine socioeconomic inequalities in HPV-related knowledge and vaccination and identified factors associated with such inequalities.

**Methods:**

Self-administered questionnaires measuring HPV-related knowledge and vaccine uptake were completed by 1,306 women through online survey platform. HPV knowledge was assessed using a 12-item question stem that covered the hazards of HPV infection, HPV vaccine dosage, benefits, and protection. Cluster analysis by combining monthly household income, educational level, and employment status was used to identify socioeconomic status (SES) class. The concentration index (CI) was employed as a measure of socioeconomic inequalities in HPV-related knowledge and vaccination. Linear regression and logistic regression were established to decompose the contributions of associated factors to the observed inequalities.

**Results:**

The CI for HPV-related knowledge and vaccine uptake was 0.0442 and 0.1485, respectively, indicating the higher knowledge and vaccination rate were concentrated in groups with high SES. Education and household income made the largest contribution to these inequalities. Age, residency and cervical cancer screening were also important contributors of observed inequalities.

**Conclusion:**

Socioeconomic inequalities in HPV-related knowledge and vaccination uptake are evident in China. Interventions to diffuse HPV-related information for disadvantaged groups are helpful to reduce these inequalities. Providing low or no-cost HPV vaccination and ensuring accessibility of vaccines in rural areas are also considered to be beneficial.

## Introduction

Cervical cancer occupies the fourth position in the list of the most common cancers causing death among females all over the world. In 2020, there were approximately 604,000 new cases of cervical cancer and 342,000 deaths caused by this disease worldwide (1). To make matters worse, Global inequalities in cervical cancer incidence and mortality are persistent and linked to deprivation and low socioeconomic status (2, 3). For example, the 5-year survival rate among women diagnosed with cervical cancer was approximately 30% lower in low than in high socioeconomic census tracts (3). It is well-known that more than 99% of cervical cancer cases are implicated with human papillomavirus (HPV) infection (4). Developing prophylactic vaccines against the most HPV infection is recommended as the most effective measure to prevent and control cervical cancer. Since HPV vaccines have been licensed globally in 2006, many countries implemented HPV vaccination programmes (5). However, vaccine coverage is not sufficient and remains unequal due to a series of socio-economic factors (6).

Similar to many countries worldwide, China also faces a great challenge in addressing the issue of HPV infection. In 2016, the HPV vaccine was approved for use in women in China. However, uptake of expensive vaccines that are not included in the Chinese Expanded Program on Immunization, the female population has to be fully self-funded for vaccination (7). Additionally, the shortage of HPV vaccine supply and negative vaccination attitudes were also considered as obstacles to vaccination uptake in China. Currently, the vaccination rate for women aged 9 to 45 is especially lower than that in other countries (8).

Fundamental cause theory contends that people who are more advantaged in terms of knowledge, money, status, and beneficial social connections are better positioned to avail themselves of health-promoting resources than less advantaged people (9). Access and reception to the HPV vaccine are dependent upon personal knowledge and financial resources. It was found that vaccination varied significantly between socioeconomic status (SES) in many countries (6). What’s worse, unequal HPV vaccination coverage may in turn cause a widened disparity in the incidence of HPV-related cancer (4). Due to the excessive income gap and uneven distribution of health resources in China, socioeconomic inequalities in health care and outcomes still exist and persist (10–12). When it comes to HPV vaccination, however, we are unknown whether its distribution is unequal as well. If so, what is the degree of inequality? Currently, HPV vaccines have been introduced in China only for a short time period. In order to prevent the widening disparity of HPV infection and cervical cancer, examining inequality in accessing to HPV vaccine become extremely important.

Several factors may drive the complex inequalities in HPV vaccination. Lack of adequate knowledge of HPV was reported to be one of the essential contributors. From vaccination intention to completion, knowledge plays a large role in the decision-making process. More HPV-related knowledge is associated with increased positive attitudes toward HPV vaccination and a stronger intention to be vaccinated (13). Yet, the group with a lower SES was found to have barriers seeking and using health information, which further resulted in low health-related knowledge, and ultimate unhealthy behaviors (14, 15). In the case of HPV, SES-based disparities in knowledge might arise at an initial stage, and further affect the vaccination disparity. Therefore, in order to understand and address inequality in HPV vaccine uptake, investigating the inequality in HPV-related knowledge is a necessary prerequisite step.

Although existing studies have provided evidence of inequalities in HPV knowledge and vaccination, the majority of studies were conducted in developed countries, such as the United Kingdom and America (6, 16–18). Evidence from developing countries is scarce. Additionally, there is paucity in the published literature using a synthetised index capturing multiple socioeconomic characteristics of individuals to assess socioeconomic inequality in HPV vaccine uptake. To fill these knowledge gaps, this study aims to examine the socioeconomic inequalities in HPV knowledge and vaccine uptake in China based on the use of concentration indices (CI), and identify the extent to which various factors contribute to any observed inequalities. To our best knowledge, this is the first study to assess socioeconomic inequalities in HPV-related knowledge and vaccination using summary measures in China. Findings also provide important policy implications for reducing the disparity in the incidence of HPV-related cancer in developing counties.

## Materials and methods

### Data sources

A cross-sectional survey was conducted online for data collection. An online survey is becoming increasingly extensive and widespread, and has been demonstrated to be completely feasible, though it has a bias toward those who have access to the online platform (19). In the present study, A digital questionnaire link was generated through the ‘Wenjuanxing’ platform,^1^ a professional and popular online questionnaire survey platform in China. This platform has a large number of potential sample users to ensure the randomness of sampling and the reliability of inferences (19, 20).

We used the sample service function on this platform to invite respondents to fill in the questionnaire. Since the HPV vaccine was approved to be used only for women in China, the males were not allowed to participate in our survey. In order to ensure a better representative sample, we required the platform to invite respondents with different demographic characteristics (e.g., age, education, residential places) to participate in the survey. Before proceeding to the questionnaire, the participants were requested to read the informed consent letter and gave their consent. Informed consent was obtained from all subjects. All methods were carried out in accordance with guidelines and regulations. Participants were allowed to withdraw at any time before completing the questionnaire. Only those questionnaires without any missing answers could be submitted successfully. The surveying period was from April to May 2023. A total of 1,421 women completed the questionnaire.

### Measurements

#### Outcome variables

The primary outcomes of interest were: (1) HPV-related knowledge and (2) HPV vaccine uptake. HPV knowledge was assessed in a 12-part item that covered the hazards of HPV infection, HPV vaccine dosage, benefits, and protection. These items have already been used and tested in previous studies employed with different populations (13, 14). Each item in the questionnaire was given true and false options. When the respondent provided a correct answer, a score of 1 was given. Otherwise, a score of 0 was assigned. In total, an aggregate score for knowledge ranged from 0 to 12. A higher score indicates better knowledge. In our study, Cronbach’s alpha for this tool is 0.823 and reliability is acceptable. More details regarding items for HPV knowledge can be found in Supplementary Table S1. HPV vaccine uptake was assessed by asking a respondent whether she ever uptake the HPV vaccine or HPV shot (yes or no).

#### Socioeconomic status

Inequalities of the HPV knowledge and vaccine uptake were estimated in the study participants with different SES. Actually, SES is a multidimensional indicator, and three major SES indicators were used in previous studies: educational attainment, occupational status, and income (6, 21). To assess comprehensive SES for respondents, cluster analysis was used to identify the latent variable of SES (22, 23). In this study, we conducted the k-means clustering algorithm by combining the socioeconomic variables of monthly household income *per capita*, educational level and employment status. We then grouped the respondents into five socioeconomic clusters. According to the characteristics of each cluster (Table 1), these five clusters were divided into very low, low, medium, high and very high SES groups.

**Table 1:** Characteristics of the five socioeconomic clusters.

	Cluster 1 (*n* = 194)	Cluster 2 (*n* = 230)	Cluster 3 (*n* = 391)	Cluster 4 (*n* = 385)	Cluster 5 (*n* = 206)
Education	Junior high school or below	Senior high school	Bachelor degree	Bachelor degree	Postgraduate degree or above
Monthly household income *per capita*	<5,000 ¥	5,000–8,000 ¥	5,000–8,000 ¥	8,001–10,000 ¥	>10,000 ¥
Employment status	No	No	Yes	Yes	Yes
Classification of SES	Very low	Low	Medium	High	Very high

#### Explanatory variables

Following previous studies (6, 24), four types of explanatory variables were included in this study to empirically examine their contributions to socioeconomic inequalities in HPV knowledge and vaccination. Demographic characteristics considered in this study were age and marital status. Socioeconomic characteristics concluded educational attainment, working status and monthly income of household *per capita*. Location variables were included to capture the potential regional heterogeneity. They were geographic locations (Eastern China, Central China, and Western China) and residential areas (urban or rural). Variables related to health status and behavior were measured by self-rated health status, history of cervical cancer within family members and attendance to cervical cancer screening in the past year. More details about the definition of these measurements are provided in the Supplementary Table S2.

### Statistical analysis

#### Measuring inequality

CI was used to assess socioeconomic inequalities in HPV knowledge and vaccination. In this study, it is twice the (weighted) covariance of the HPV-related knowledge scores and vaccine uptake (y) and the relative rank of the study participants in their self-rated SES (γ), divided by the mean of the HPV-related knowledge scores and vaccine uptake. The CI formula is as follows:


$$C=\frac{2}{\mu }COV\left(y,\gamma \right)$$

The value of CI ranges between −1 and + 1. A value of zero indicates an absence of inequality, while a greater distance from zero indicates a higher level of inequality. A positive concentration index means that proper HPV knowledge and high vaccine coverage are concentrated among the relatively higher SES, and vice versa (25).

#### Decomposing inequality

In order to understand the contribution of each explanatory variable to the observed inequalities, we also followed the method proposed by Wagstaff et al. (26) to decompose CI. Firstly, we established a regression model on the outcome variable (y):


$$y_{i}=a^{m}+\underset{k}{\sum }\beta_{k}^{m}x_{ki}+\mu_{i}$$

Where $\beta_{k}^{m}$ is the marginal effect (dy/dx) of each explanatory variable x; $\mu_{i}\;$ indicates the error term.

In this analysis, a linear regression model for HPV knowledge and logistic regression for HPV vaccine uptake was employed.

The CI for y can then be expressed as follows:


$$C=\underset{k}{\sum }\left(\beta_{k}\bar{x_{k}}/\mu \right)c_{k}+GC_{\varepsilon }/\mu$$

Where C is the concentration index of the outcome variable (y); $\beta_{k}$ is the marginal effect of $\;x_{k}$ ; $\bar{x_{k}}\;$ and $c_{k}\;$ are the mean and the concentration index of $\;x_{k}$ ; μ is the mean of y; $GC_{\varepsilon }$ is the generalized concentration index for ε. The total CI is made up of two components: explained component ( $\underset{k}{\sum }\left(\beta_{k}\bar{x_{k}}/\mu \right)c_{k}$ ) and residual component ( $GC_{\varepsilon }/\mu$ ). The explained component actually reveals that the contribution of each explanatory variable to inequality is calculated according to the interaction between the elasticity of the outcome variable ( $\beta_{k}\bar{x_{k}}/\mu$ ) with respect to that variable and socioeconomic inequality in the distribution of the variable ( $c_{k}$ ) (26).

All data management and statistical analysis were performed on STATA 16.0 and a *p*-value of less than 0.05 was considered to be statistically significant.

## Results

### Characteristics of study participants

After the exclusion of the returned questionnaires containing logic errors, a final sample size of 1,306 (91.9%) was used for data analysis. In study participants, the most of females aged between 21 to 40 (76.3%) and got married (66.8%). The respondents were well educated, with 62.7% having a bachelor degree. 35.1% of respondents had a monthly household income *per capita* greater than 10,000 yuan. More than half of the respondents (62.9%) resided in urban. A small portion of the family members of respondents (9.3%) had a history of cervical cancer. More than a quarter of women (32.6%) attended cervical cancer screening in the past year (Table 2).

**Table 2:** Characteristics of study participants.

Explanatory variables	N	%	HPV-related knowledge		HPV vaccine uptake	
			Mean (S.D.)	*p*	*N* (%)	*p*
Total			9.57 (1.99)		621 (47.5)	
Age (year)						
≤20	55	4.2	9.44 (2.06)	<0.001	15 (1.1)	<0.001
21–30	508	38.9	10.20 (1.51)		304 (23.3)	
31–40	489	37.4	9.69 (1.77)		338 (25.9)	
41–50	161	12.3	8.14 (2.45)		45 (3.4)	
≥51	93	7.1	8.08 (2.41)		19 (1.5)	
Marital status						
Married	872	66.8	9.32 (2.11)	<0.001	522 (40.0)	<0.001
Others	434	33.2	10.07 (1.59)		199 (15.2)	
Education						
Junior high school or below	128	9.8	7.30 (2.10)	<0.001	5 (0.4)	<0.001
Senior high school	97	7.4	7.44 (2.63)		33 (2.5)	
Bachelor degree	819	62.7	10.07 (1.54)		492 (37.7)	
Postgraduate degree or above	262	20.1	9.90 (1.58)		191 (14.6)	
Monthly household income *per capita* (¥)						
<5,000	341	26.1	9.12 (2.21)	<0.001	106 (8.1)	<0.001
5,000–8,000	287	22.0	9.55 (2.15)		135 (10.3)	
8,001–10,000	220	16.8	9.80 (1.83)		134 (10.3)	
>10,000	458	35.1	9.81 (1.71)		346 (26.5)	
Employment status						
No	168	12.9	8.85 (2.07)	<0.001	43 (3.3)	<0.001
Yes	1,138	87.1	9.68 (1.95)		678 (51.9)	
Residency						
Urban	821	62.9	9.82 (1.85)	<0.001	570 (43.6)	<0.001
Rural	485	37.1	9.16 (2.13)		151 (11.6)	
Geographic location						
East	777	59.5	9.45 (2.10)	0.010	443 (33.9)	0.003
Central	388	29.7	9.68 (1.88)		219 (16.8)	
West	141	10.8	9.96 (1.53)		59 (4.5)	
History of cervical cancer						
No	1,181	90.7	9.02 (2.06)	0.001	615 (47.1)	<0.001
Yes	121	9.3	9.63 (1.97)		106 (8.1)	
Self-rated health status						
Bad	67	5.1	8.75 (2.12)	<0.001	18 (1.4)	<0.001
Fair	294	22.5	9.28 (2.25)		116 (8.9)	
Good	945	72.4	9.72 (1.86)		587 (44.9)	
Cervical cancer screening						
No	880	67.4	9.50 (2.08)	0.050	402 (30.8)	<0.001
Yes	426	32.6	9.73 (1.77)		319 (24.4)	

In terms of outcomes, the average HPV-related knowledge scores were 9.57 ± 1.99, and 47.5% of respondents reported they have been vaccinated. Additionally, there were significant differences in knowledge and vaccination uptake among respondents with different characteristics (Table 2). For example, high school graduates had lower scores in HPV-related knowledge than those with higher education level (*p* < 0.001).

### Inequalities in HPV-related knowledge and vaccine uptake

**Figure 1:**
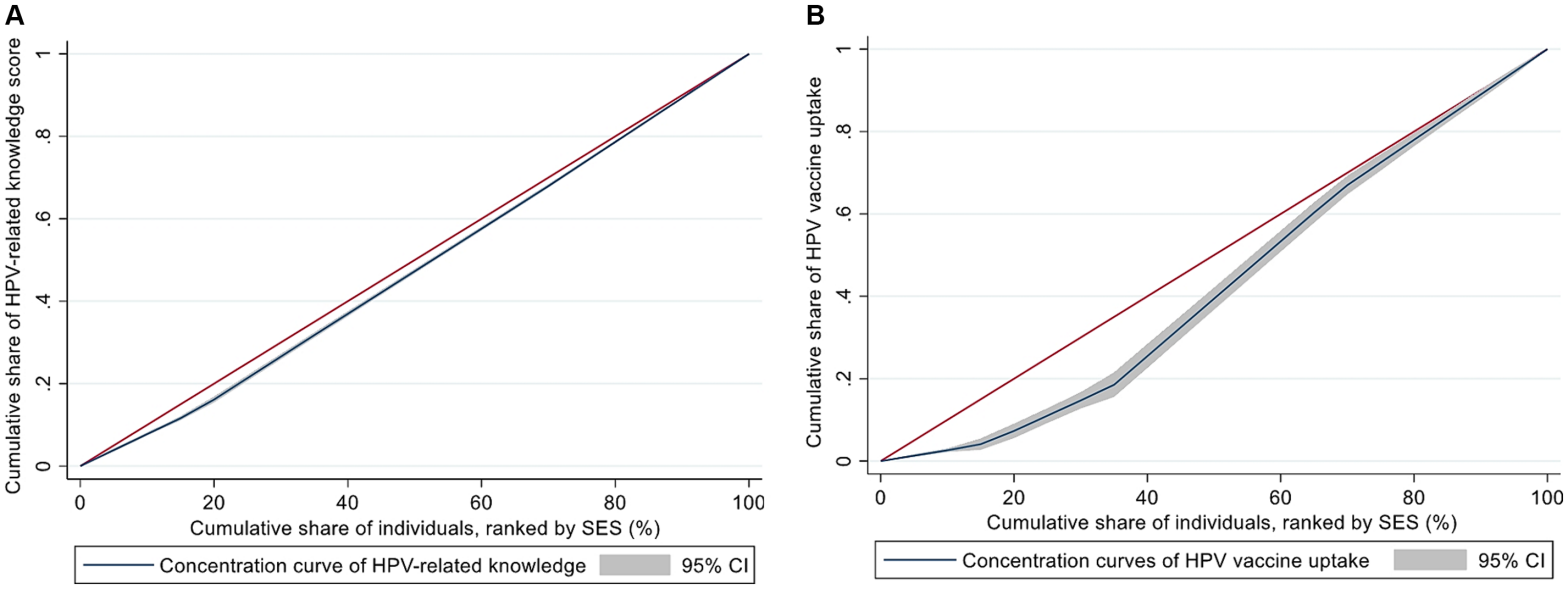
Concentration curves of the HPV-related knowledge **(A)** and vaccine uptake **(B)**. The red line represents the line of absolute equality and the blue line is the concentration curve.

Figure 1 showed the concentration curve of HPV-related knowledge and vaccine uptake. Both curves lay under the 45-degree line (the line of absolute equality), and the corresponding concentration index was 0.0442 for HPV-related knowledge and 0.1485 for HPV vaccine uptake. The results indicate that a piece of higher HPV-related knowledge and vaccination rate were more concentrated in those respondents who had a high SES.

### Decomposition of inequalities in HPV-related knowledge and vaccine uptake

**Figure 2:**
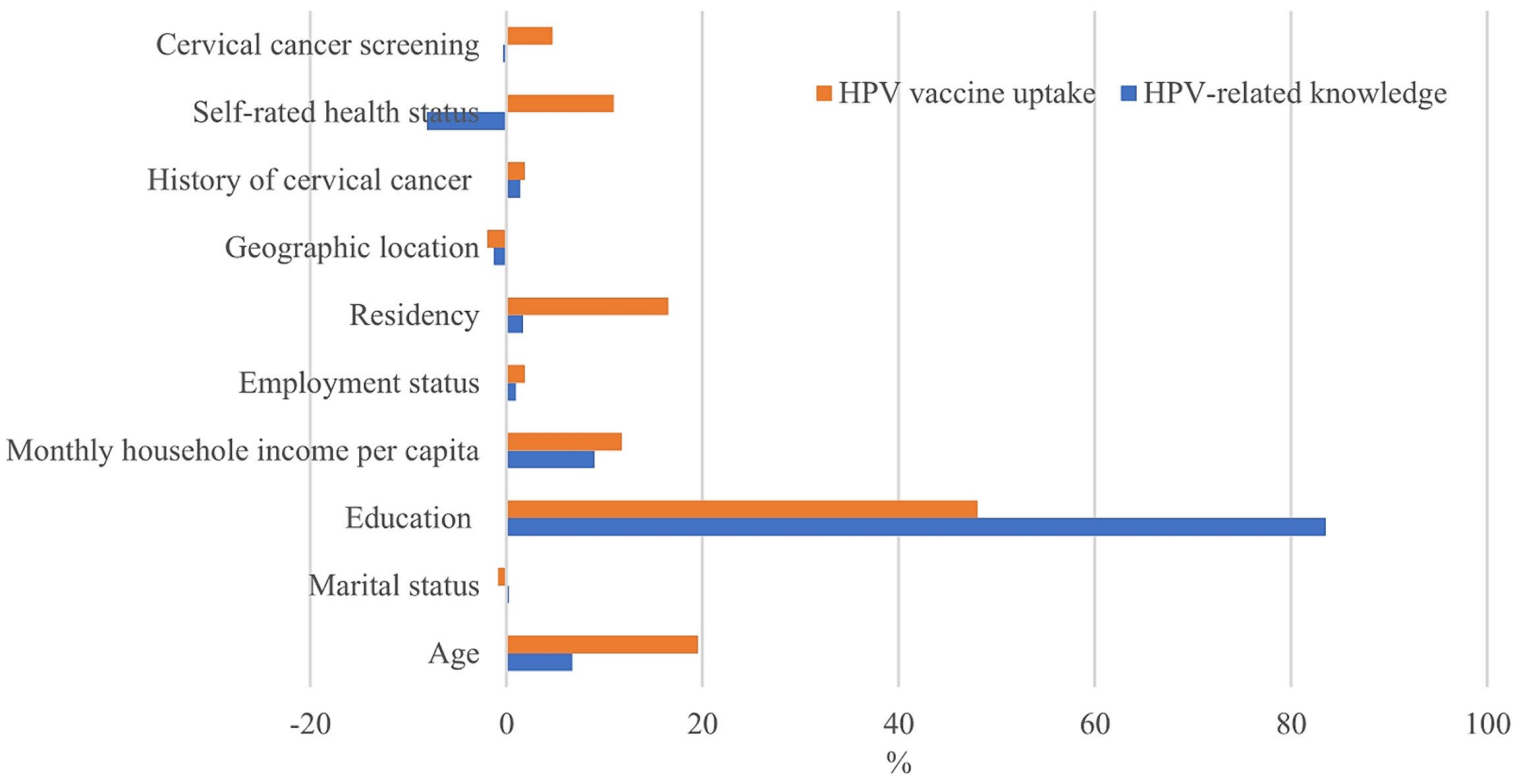
Contribution of explanatory variables to inequalities in HPV-related knowledge and vaccine uptake.

The decomposition results on the CI of HPV-related knowledge and vaccine uptake were provided in Table 3. The aggregate percentage contribution of each explanatory variable was presented in Figure 2. Since the outcome variables of interest were concentrated among the group with high SES, the positive contribution of variables means that these variables increase the degree of observed inequalities. Education was found to make the biggest contribution to socioeconomic inequalities in HPV-related knowledge (83.55%) and vaccine uptake (48.04%). Household income and age were also significant contributors of the observed inequalities. Residing in the urban and attendance to cervical cancer screening, respectively, contributed 16.53 and 4.71% to the unequal distribution of HPV vaccine uptake, whereas their contributions to inequality in knowledge regarding HPV were very small. Notably, the contribution of self-rated fair and good health status to the knowledge inequality was negative (−8.10%), but its contribution to the vaccination inequality was positive (10.95%).

**Table 3:** Decomposition of CI of HPV-related knowledge and vaccine uptake.

	C_k_	HPV-related Knowledge			HPV vaccine uptake		
		Margin	Absolute contribution	Percentage contribution	Margin	Absolute contribution	Percentage contribution
Age (ref. = ≤20)							
21–30	0.125	0.402	0.0021	4.63	0.123	0.0109	7.34
31–40	0.134	0.186	0.0009	2.18	0.007	0.0006	0.42
41–50	−0.433	−0.093	0.0005	1.14	−0.099	0.0093	6.29
≥51	−0.588	0.125	−0.0005	−1.21	−0.109	0.0081	5.49
Marital status (ref. = married)							
Others	0.005	0.184	0.0001	0.28	−0.102	−0.0012	−0.83
Education (ref. = Junior high school or below)							
Senior high school	−0.803	−0.022	0.0001	0.29	0.300	−0.0306	−20.64
Bachelor degree	0.190	2.486	0.0311	70.34	0.386	0.0841	56.57
Postgraduate degree or above	0.117	2.336	0.0057	12.92	0.423	0.0179	12.11
Monthly household income *per capita* (ref. = <5,000)							
5,000–8,000	0.281	0.259	0.0017	3.78	0.041	0.0046	3.10
8,001–10,000	0.527	0.234	0.0022	4.95	0.071	0.0115	7.78
>10,000	0.019	0.167	0.0001	0.26	0.109	0.0013	0.88
Employment status (ref. = no)							
Yes	0.057	0.083	0.0004	0.97	0.031	0.0027	1.88
Residency (ref. = urban)							0
Rural	−0.062	−0.085	0.0008	1.71	−0.159	0.0245	16.53
Geographic location (ref. = east)							0
Central	0.074	−0.181	−0.0004	−0.94	−0.016	−0.0006	−0.43
West	0.028	−0.122	−0.0001	−0.33	−0.110	−0.0022	−1.54
History of cervical cancer (ref. = no)							
Yes	0.073	0.901	0.0006	1.39	0.237	0.0028	1.90
Self-rated health status (ref. = bad)							
Fair	0.169	−0.379	−0.0015	−3.48	0.052	0.0036	2.47
Good	0.077	−0.353	−0.0020	−4.62	0.125	0.0126	8.48
Cervical cancer screening (ref. = no)							
Yes	0.124	−0.033	−0.0001	−0.31	0.094	0.0069	4.71

## Discussion

This study provides the first empirical evidence on the existence of socioeconomic inequalities in HPV-related knowledge and vaccine uptake in China. The distribution of knowledge and vaccination tend to bias toward the groups with high SES. These results are consistent with the findings of studies conducted in some other countries (6). Since the HPV vaccine was introduced for a short time in China, vaccines are insufficient, and relevant educational programs have not been widely implemented. Under such circumstances, persons from more (versus less) advantaged SES groups can make better use of their social resources to access to vaccines and benefit themselves greatly (9). If the disproportionate distribution of vaccination still continues, ultimately, the disparity in HPV-attributable cancer incidence is expected to be widen.

Among those key factors contributing to the observed inequalities, educational attainment is the biggest contributor. People with a higher level of education are more likely to have better health literacy (27). They usually actively seek information on HPV infection and vaccines from various sources, and have a better ability to understand complex information and judge the accuracy of information (28). Previous studies suggested that a higher level of HPV-related knowledge had an association with increased intention and acceptance to be vaccinated (29–31). It is therefore observed that the well-educated population have high HPV vaccination uptake. Although many women appear to have heard of HPV in China, A small portion of people understand the HPV vaccine and its effects. A health education campaign should be carried out to address deficiencies in HPV knowledge for individuals with low education. Additionally, Precaution Adoption Process Model underscores the importance of response efficacy (belief in the effectiveness of the recommended action) and self-efficacy (confidence in one’s ability to perform the action) in the stages of going through from lack of awareness to the adoption of a precautionary behavior (32). While knowledge dissemination is critical, fostering a belief in the effectiveness of health behaviors and enhancing individuals’ confidence in their ability to undertake HPV vaccine are equally vital.

The contribution of household income remained to be considerably significant after controlling for other explanatory variables. Currently, the HPV vaccine is not provided free of charge and medical insurance cannot cover HPV vaccine costs as well in China (33). Being vaccinated for the three doses costs at least USD 360 (¥2,397 RMB), which is beyond the range of affordability for many women and families in China (8). Obviously, poor population are less likely to complete vaccination, resulting in low attention to HPV-related information (24). In order to realize a high vaccine uptake in China, it is particularly helpful to implement a national HPV immunization program and provide low or no-cost HPV vaccination.

In addition to socio-economic factors, we also found that a certain share of the observed inequalities is explained by age. Specifically, young women have better HPV-related knowledge and a higher possibility of being vaccinated. On the one hand, young people generally have a better ability to learn and accept new knowledge than their old counterparts (34). Knowledge regarding HPV is not exceptional. On the other hand, young people have a better immunogenic response than old people (35). Health Belief Model posits that health behaviors are influenced by individuals’ perceptions of susceptibility, severity, benefits, and barriers related to a disease (36, 37). For instance, perceived benefits and barriers significantly impact decision-making processes. To encourage vaccination as early as possible, the information on higher HPV vaccine effectiveness when vaccinated at a young age is widely disseminated to the public in China (38). This further stimulates the female at a young age to initiate and complete vaccination.

Residency may play a role in explaining the inequality of HPV vaccine uptake. This finding may be attributed to significant urban–rural differences in social and economic circumstances in China. A large number of medical facilities and skilled medical practitioners are concentrated in urban areas, which led to a significant urban–rural disparity in access to health care (39, 40). In China, vaccines are currently in shortage and only available in urban areas, which undoubtedly generated many obstacles for rural residents to access to vaccines (8). As such, ensuring the accessibility of vaccines to rural women needs to be given priority in China.

In line with other studies (41, 42), we also found that unequal distribution of cervical cancer screening made some contributions to the inequality in vaccination uptake. As one of the important cervical cancer preventive measures, the female population who attend screening regularly are considered to have positive attitudes, beliefs or values regarding preventive health care, better perceptions of the risk of HPV infection, and also pay more attention to their own health. Combining these potential factors, it is not difficult to understand the occurrence of decision-making behavior for being vaccinated. Additionally, during performing cervical cancer screening, a physician might provide more information on HPV vaccines for women and encourage them to participate in vaccination uptake.

Interestingly, contributions of self-rated health status to inequalities of HPV knowledge and vaccination were the opposite. A previous study found personal perception of health affected certain health behaviors (43). The females with self-rated good health tend to make greater active and successful attempts at maintaining a healthy lifestyle, and knew what it took to be healthy as well. These people, therefore, have a stronger intention to be vaccinated against HPV infection. However, this factor made a negative contribution to socioeconomic inequality in HPV knowledge. People with perceived poor health status have more tendency to search for health-related information in comparison to healthy individuals, which further increased the possibility of exposure to HPV-related information (44, 45). As a result, the socioeconomic inequality in HPV-related knowledge was slightly offset by this negative effect of health status.

Some limitations in this study should be acknowledged. First, due to the cross-sectional design used in this study, the temporality and causality of the observed relationships cannot be explored. Second, several individual-level variables included in this study were used to explain the observed inequalities. Those variables at the household and regional levels that may have potential effects on HPV knowledge and vaccination were not considered due to the availability of data. Third, a new survey mode was adopted through an online platform. Although we have used the sample service function to improve sample representativeness, only individuals who are interested in our topic filled out the questionnaire. Those who are unable to access the internet and have low levels of education are unlikely to participate in the survey. This led to our sample with a high level of education and being young.

## Conclusion

Strong socioeconomic inequalities in HPV-related knowledge and vaccine uptake exist in China. Income and education make the greatest contribution to these inequalities. Additionally, other factors, such as age, residency and cervical cancer screening, are also important contributors. In order to reduce these inequalities, it is recommended to implement a health education campaign to diffuse HPV-related information for disadvantaged groups. Additional policy implications from our findings lie on providing low or no-cost HPV vaccination and ensuring accessibility of vaccines for these hard-to-reach women in rural areas.

## Data availability statement

The raw data supporting the conclusions of this article will be made available by the authors, without undue reservation.

## Ethics statement

The studies involving humans were approved by the Ethics Committee of Yuebei People’s Hospital (approval number: KY-2022-048). The studies were conducted in accordance with the local legislation and institutional requirements. The participants provided their written informed consent to participate in this study.

## Author contributions

XG: Writing – original draft, Conceptualization. JX: Methodology, Software, Writing – original draft. YH: Writing – review & editing. GZ: Methodology, Writing – review & editing. JL: Conceptualization, Funding acquisition, Writing – review & editing.

## Footnotes

^1^ https://www.wjx.cn
