# Supplementary material for: Socioeconomic inequalities in human papillomavirus knowledge and vaccine uptake: evidence from a cross-sectional study in China
Source: Front Public Health. 2024 Jun 27;12:1399192. doi: 10.3389/fpubh.2024.1399192 (PMC11236539; doi:10.3389/fpubh.2024.1399192)
Supplement: Supplementary file 1 [file Table_1.DOCX]

**Supplementary file 1**

**Table S1 Measurement items for HPV-related knowledge**

| **Items** | **Mean (S.D.)** |
| --- | --- |
| 1. HPV is a rare infection | 0.72 (0.49) |
| 2. HPV is a sexually transmitted infection (STI) | 0.89 (0.31) |
| 3. Infection with HPV can lead to cervical cancer | 0.88 (0.32) |
| 4. Men cannot get HPV | 0.66 (0.42) |
| 5. HPV has many different types | 0.96 (0.20) |
| 6. HPV infection can lead to AIDS | 0.63 (0.48) |
| 7. HPV infection can be cured with antibiotics | 0.70 (0.45) |
| 8. The HPV vaccines offer protection against all sexually transmitted infections | 0.60 (0.49) |
| 9. Someone who has had the HPV vaccine cannot develop cervical cancer | 0.96 (0.19) |
| 10. One of the HPV vaccines offers protection against genital warts | 0.78 (0.42) |
| 11. Girls who have had the HPV vaccine do not need a Pap smear test when they are older | 0.95 (0.22) |
| 12. HPV vaccines require more than one dose | 0.94 (0.23) |

**Table S2 Definition and measurements of explanatory variables**

| **category** | **Variable name** | **Type of variable** | **Measurements** |
| --- | --- | --- | --- |
| Demographic  variables | Age (year) | Categorical:  1= ≤20  2= 21-30  3= 31-40  4= 41-50  5= ≥51 | Self-reported by respondents at the time of the survey |
|  | Marital status | Categorical:  1= Married  2= Others | Current marital status reported by respondents at the time of the survey |
| Socioeconomic variables | Education | Categorical:  1=Junior high school or below  2=Senior high school  3=Bachelor degree  4=Postgraduate degree or above | The highest level of education reported by a respondent |
|  | Monthly household income per capital (¥) | Categorical:  1=＜5000  2=5000-8000  3=8001-10000  4=＞10000 | Monthly household income per capital reported by a respondent |
|  | Employment status | Categorical:  0= No  1= Yes | Whether a respondent was employed at the time of the survey |
| Location variables | Residency | Categorical:  1=Urban  2=Rural | Current residential area of respondent |
|  | Geographic location | Categorical:  1=East  2=Central  3=West | Which region of China does the respondent reside in |
| Health and health-related behaviour | History of cervical cancer | Categorical:  0=No  1=Yes | Does the respondent's family member have a history of cervical cancer |
|  | Self-rated health status | Categorical:  1=Bad  2=Fair  3=Good | Self-rated health status in the past six months reported by a respondent |
|  | Attendance to cervical cancer screening | Categorical:  0=No  1=Yes | Has the respondent participated in cervical cancer screening in the past year |
